# Emotional Responses in Clinical Ethics Consultation Decision-Making: An Exploratory Study

**DOI:** 10.3390/bs15060748

**Published:** 2025-05-29

**Authors:** Margherita Dahò

**Affiliations:** Department of Psychology, Educational Sciences and Human Movement, University of Palermo, Viale Delle Scienze Ed. 15, 90128 Palermo, Italy; margherita.daho@unipa.it

**Keywords:** decision-making, moral reasoning, emotion, cognitive psychology, medical ethics, clinical ethics, clinical ethics consultants, bioethics, healthcare

## Abstract

Integrating Clinical Ethics Consultants (CECs) into healthcare requires understanding how they apply bioethical knowledge while managing cognitive and emotional challenges in ethical deliberations. Ethical consultations often elicit strong emotions, yet their impact on decision-making remains underexplored. This study explores the emotional responses of 52 CECs from the United States and 10 European countries through a semi-structured survey. Participants selected a real ethical case they had encountered and described their emotional reactions during and after deliberation. Findings revealed that almost 77% of CECs experienced negative emotions such as frustration, sadness, or anger during deliberation, while 21% reported neutral or positive feelings. Although satisfaction and relief increased after deliberation, negative emotions often persisted. Additionally, 45% of participants reported feelings of inadequacy or remorse, 12% expressed uncertainty about their decision, and 5% stated they would change their decision in hindsight. The accumulation of negative emotions may affect CECs’ well-being and judgment, highlighting the need for structured support. Managing cognitive and emotional demands is essential to maintaining CECs’ effectiveness, underscoring the importance of targeted training programs and support strategies to enhance ethical decision-making and resilience in high-stakes medical contexts.

## 1. Introduction

Bioethics focuses on morally appropriate decisions in healthcare and life sciences (Beauchamp & Childress, 2013). Often equated with clinical ethics, it provides principles for clinical practice and research (Varkey, 2021). While some scholars view bioethics as influenced by American cultural values (Jonsen, 1998), Gracia (2001) highlights broader influences, including Western secularization and autonomy in life-and-death decisions. Ethics is deeply embedded in community and culture, reflecting individual and collective values (Callahan, 1994). Indeed, cross-cultural differences in ethical perspectives among healthcare professionals can lead to conflicts (Oberle & Hughes, 2001). Beauchamp and Childress (2013) define Anglo-American bioethics around four principles: autonomy, beneficence, non-maleficence, and justice. In contrast, European bioethics varies due to historical, cultural, and socio-political factors (Welie & Ten Have, 1992). In Mediterranean contexts, Leone (1990) describes a “realistic” and “personalist” approach, emphasizing trust and personal responsibility over strict autonomy. Central European ethics reflect Enlightenment ideals of rights and duties, whereas Eastern European bioethics, shaped by totalitarian regimes, often prioritized collective needs over individual rights (MacIntyre, 1984; Orzechowski et al., 2020). Theories of moral pluralism and monism thus play a significant role in shaping the ethical landscape. Pluralism acknowledges multiple, potentially conflicting moral perspectives, while monism advocates for a unified, overarching moral principle (Varkey, 2021). This tension is particularly relevant in the practice of Clinical Ethics Consultants (CECs), where ethical dilemmas often involve diverse cultural, religious, and personal beliefs. The influence of these differing worldviews can significantly affect how CECs navigate ethical deliberations, requiring sensitivity to both rational and emotional factors.

### 1.1. The Development, Nature, and Goals of Clinical Ethics Consultation

CECs play a crucial role in ethical decision-making within healthcare, mediating complex cases including birth and end-of-life care, informed consent, and treatment refusal. Unlike institutional ethics committees, which focus on policy guidance, CECs may engage directly with healthcare teams, patients, and families to address ethical dilemmas in real time (Picozzi & Gasparetto, 2020). The formal use of ethics committees began with the 1976 Quinlan case, when the New Jersey Supreme Court recommended an ethics committee to assess a comatose patient’s prognosis (Fleetwood et al., 1989). This decision spurred the creation of institutional ethics committees. In the US, the prevalence of ethics committees rose from 1% of hospitals in 1982 to 60% by 1987 (Aulisio, 2016; Celie & Prager, 2016), with consultations increasing by 94% by 2000 (Fox et al., 2022). In Europe, their development has varied: Norway mandated them in major hospitals from 1996, Belgium required them in all general and psychiatric hospitals from 1994, and Italy expanded their scope in 2006 (Hajibabaee et al., 2016; Galván Román et al., 2021). These committees support healthcare providers, mediate disputes, and improve patient care (Alexander et al., 2024; Engelhardt, 2011; Fiester, 2024; Ilse et al., 2021; Picozzi & Gasparetto, 2020). They also guide ethical decision-making and help prevent conflicts.

CECs conduct ethics consultations through a structured process. When an ethical dilemma arises, a consultation is initiated by a healthcare provider, patient, or family member. The consultant gathers relevant clinical, legal, and contextual information, reviews medical records, and speaks with stakeholders. They then facilitate discussions, ensuring all perspectives are considered and ethical principles are addressed (Alexander et al., 2024; Engelhardt, 2011; Fiester, 2024; Ilse et al., 2021; Picozzi & Gasparetto, 2020). Finally, CEC can clarify value conflicts, interpret advance directives, determine decision-making capacity, and resolve disagreements. While they do not impose decisions, their analysis structures, deliberates and helps reach ethically sound conclusions. Therefore, given the complexity of the role, CECs require specialized competencies. According to the American Society for Bioethics and Humanities (ASBH, 2011), these fall into three main categories:

- *Ethical Assessment and Analysis*: Ethical reasoning, understanding moral concepts, and identifying value conflicts.
- *Process Skills*: Organizing discussions, self-assessment, and quality improvement.
- *Interpersonal Skills*: Communication, active listening, and cultural sensitivity. Moreover, humility, prudence, compassion, honesty, and patience are critical for building trust with stakeholders (Lipman & Powell, 2016).

Beyond these core competencies, CECs require problem-solving, decision-making, and leadership skills. They must understand ethical theories and be familiar with institutional policies on informed consent, pain management, and organ donation (Picozzi & Gasparetto, 2020). Additionally, CECs contribute to institutional ethics policies, staff education, and proactive ethics interventions. They develop guidelines for handling recurring ethical issues, train healthcare teams, and support quality improvement initiatives.

These competencies and duties must be viewed through a lens recognizing the dynamic interaction between emotional intuition and rational deliberation. Emotions, often seen as subjective, are becoming increasingly acknowledged as fundamental in CEC practice. They may affect ethical judgments, particularly in emotionally charged situations (Conway & Gawronski, 2013; Helion & Ochsner, 2018). For instance, the decision to withhold life-sustaining treatment may be influenced by both ethical principles and the emotional responses of the healthcare team and the family.

### 1.2. Decision-Making and Moral Reasoning Theories

Moral reasoning is central to ethical decision-making. Kohlberg’s rationalist model aligns with Kantian ethics, positing that moral decisions arise from deliberate reasoning. However, Haidt’s socio-intuitionist model (Haidt, 2001; Haidt & Bjorklund, 2007) suggests that moral judgments are primarily intuitive, with reasoning used post hoc to justify gut responses. This view builds on Hume’s emphasis on moral emotions. Though Haidt’s model is criticized for downplaying cultural influences, research supports the role of emotions in moral judgment and decision-making (Conway & Gawronski, 2013; Gangemi et al., 2025; Greene & Haidt, 2002; Haidt & Bjorklund, 2007; Helion & Ochsner, 2018).

Contemporary theories integrate intuitive–emotional and rational–cognitive processes. For instance, Greene et al. (2001, 2004, 2008), Greene (2005) and Greene and Haidt (2002), propose a dual-process theory: deontological judgments arise from emotional responses, while utilitarian decisions require cognitive control. For instance, studies indicate that individuals with strong working memory favor utilitarian choices (Moore et al., 2008), whereas rapid, instinctive responses align with deontological reasoning (Suter & Hertwig, 2011; Zhang et al., 2017). Furthermore, it has been shown that moral dilemmas often provoke emotions like disgust and anger (Avramova & Inbar, 2013; Horne & Powell, 2016). Finally, neuroimaging studies link controlled reasoning to the prefrontal cortex and emotional responses to the amygdala (Pascual et al., 2013; Helion & Ochsner, 2018).

However, despite these insights, the emotional impact on moral reasoning is still debated. Christensen and Gomila (2012) suggest that factors like the framing of dilemmas—whether options are presented as “saving” or “killing”—can significantly affect moral judgments (see also “Framing Effect” theory, Tversky & Kahneman, 1981). Other studies link lower empathy to utilitarian decision-making (Gleichgerrcht & Young, 2013; Wiech & Tracey, 2013), while Białek and De Neys (2017) argue that utilitarian judgments can be automatic, supporting Johnson-Laird’s (2010) Mental Models theory. Finally, Kahneman and Egan (2011) distinguish “fast” intuitive (System 1) and “slow” rational (System 2) thinking. While fast thinking aids in clinical emergencies, slow thinking is crucial for ethical dilemmas, though biases can still influence reasoning (Blumenthal-Barby & Krieger, 2015; Kahneman & Egan, 2011; Whelehan et al., 2020). This integration of emotional and rational elements is critical for CECs, whose role requires them to navigate between these different modes of reasoning. For example, while a quick, intuitive judgment might suggest a course of action in an urgent clinical situation, a slower, more deliberate rational process is required when considering the long-term implications of ethical decisions. The tension between these two forms of moral reasoning—intuitive vs. rational—poses unique challenges for CECs, who must balance the immediacy of emotions with the need for careful ethical analysis.

### 1.3. The Current Study

As CECs play an essential role in healthcare decision-making, understanding how they integrate bioethical knowledge with cognitive and emotional processes is critical. Research suggests inadequate ethical decision-making can negatively impact patient outcomes (Preisz, 2019); for instance, failure to appropriately address ethical concerns in end-of-life care may lead to prolonged suffering or unwanted aggressive treatment.

Previous studies have primarily relied on Kohlberg’s rationalist model (Racine, 2008; Self & Skeel, 1991, 1998; Tsai et al., 2009), but emerging evidence suggests that consultants often make intuitive decisions and later rationalize them (Wasserman et al., 2015). Biases, institutional pressures, and personal beliefs also influence consultants’ deliberation (Albisser Schleger et al., 2011; Hofmann, 2023; Magelssen et al., 2014; Marcus, 2017; Pedersen et al., 2009a, 2009b; Preisz, 2019; Räsänen, 2023; Rogerson et al., 2011).

On the other hand, research on how emotions shape ethical reasoning in consultations remains limited. Identifying the types of emotions experienced by CECs is thus necessary to understand their impact on moral decision-making. Spronk et al. (2022) conducted an initial study on this topic with a small sample size (12 consultants), highlighting the need for further research. This study thus expands on existing research by analyzing the range of emotions experienced by CECs during and after moral deliberation. A semi-structured survey was distributed online to CECs in the U.S. and Europe to capture their emotional responses, doubts, and reflections. Given the emotionally charged nature of ethics consultations involving vulnerable individuals (Pochard et al., 2001; Spronk et al., 2022), understanding these emotional influences is crucial for improving ethical decision-making processes.

## 2. Materials and Methods

### 2.1. Data Collection

Participants were personally contacted in English, French, or Spanish, using details obtained from various online platforms, including hospital, university, and association websites that listed medical ethics consultation services (e.g., Eacmeweb.com, Ukcen.net). Approximately 80 ethics consultants were reached via email. Data were collected anonymously through Google Forms, ensuring that participants’ email addresses remained confidential.

### 2.2. The Semi-Structured Survey

Initially, demographic data such as gender, country of origin, years of service, and highest level of education (including the field) were collected, along with consent to participate in the study. The semi-structured survey consisted of 8 open-ended questions (see Table 1). To ensure inclusivity and gather comprehensive insights from non-English-speaking consultants, an official translator translated the survey into Spanish and French.

The first question prompted CECs to select and explain a clinical case they encountered and perceived as “challenging”. The term “challenging” was deliberately chosen for its ambivalence, as it can mean both “stimulating” or “interesting” as well as “puzzling” or “demanding”. This choice aimed to prevent any influence on case selection that might arise from more specific terms like “easy”, “complex”, or “recent” (Christensen & Gomila, 2012). Participants thus had the opportunity to describe their experiences in a way that captured their nature without necessarily labeling them as negative or positive. On the other hand, asking to report a standard, simple, or everyday case would probably not have triggered vivid recollections. Additional detailed questions were incorporated to gain insight into the deliberation process of this case and assist participants in recalling their most vivid memories.

Next, CECs were asked open-ended questions about the emotions they experienced during and after case deliberation. To avoid influencing responses, no specific emotions were listed. Some might argue that using a validated measure like the PANAS (Positive and Negative Affect Schedule; Watson et al., 1988) would have facilitated memory recall. However, given the study’s exploratory nature, structured response options could have introduced bias (Elston, 2021). Selecting from predefined categories may shape recollections in ways that do not fully capture participants’ actual emotional experiences. Instead, allowing respondents to describe their sensations freely promotes authenticity and spontaneity, capturing a broader spectrum of emotions, including complex or mixed feelings (Oranga & Matere, 2023). However, this approach also has limitations. Participants may not recall or articulate every emotion they experienced, a common challenge in qualitative research. Additionally, not all individuals may be accustomed to identifying and labeling their emotions. Nevertheless, ethics consultants are expected to develop emotional awareness through training and practice. They often assess patients’ cognitive and emotional states or provide support to healthcare professionals (Chooljian et al., 2016). Given these responsibilities, it is reasonable to assume that CECs possess introspective abilities and can accurately report their emotional experiences.

The final three questions explored feelings of regret or remorse regarding the case chosen for the study. Unlike other emotions, regret is especially sensitive because it may involve critical self-reflection and admitting past mistakes (Boyle et al., 2020). This can be emotionally challenging, leading individuals to avoid discussing regrets to protect themselves from negative self-evaluation or external judgment, even in an anonymous survey (Boyle et al., 2020). To mitigate this, participants were first asked whether they wished they had acted differently if given the opportunity. This open-ended phrasing encouraged reflection without explicitly mentioning “regret” or “remorse”, reducing potential bias. The second question directly addressed whether consultants experienced regret about the case, while the final question asked if they would reaffirm or change their decision.

### 2.3. Data Analysis

#### 2.3.1. Demographic Data

Demographic data were analyzed using SPSS 20, with frequency analysis conducted to summarize key characteristics.

#### 2.3.2. Qualitative Analysis of Open-Ended Responses

Open-ended survey responses were analyzed using a structured mixed-method approach. According to Cortini and Tria (2014), three primary methods are used to examine textual or narrative data:

- Narrative Style Analysis—Focuses on metaphor use and distinctive terminology.
- Word Frequency and Associations—Examines the prevalence and connections between words.
- Integrated Qualitative–Quantitative Analysis—Combines both perspectives for a comprehensive evaluation.

This study adopted the third approach, integrating word frequency analysis with a thematic analysis performed following Clarke and Braun’s (2017) guidelines without software assistance. The six-step process that the author of this paper conducted included the following:

1. Reviewing the dataset to gain familiarity with key ideas.
2. Systematically coding data segments with concise descriptors.
3. Identifying patterns among coded segments to develop themes.
4. Refining themes for coherence and distinctiveness.
5. Assigning clear labels to themes, supported by illustrative quotes.
6. Having an external expert in qualitative analysis who reviewed the final themes for consistency and alignment with research objectives.

#### 2.3.3. Categorization of Clinical Case Descriptions

Following Clarke and Braun’s method for thematic analysis (Clarke & Braun, 2017), clinical ethics consultation cases were initially reviewed and grouped according to their primary medical condition or ethical concern (e.g., end-of-life decisions, maternal–fetal conflicts, refusal of treatment, etc.). This preliminary categorization provided a structured framework for interpreting each case’s clinical and ethical complexity. Categories were generated through an inductive coding process, guided by the semantic content of the consultations. Cluster names were then assigned to reflect both the conceptual focus and internal consistency of the grouped cases. Subsequently, a second level of thematic coding focused on the ethical questions raised during the consultations. This included analyzing the nature of the moral dilemma, such as decisions around initiating, withholding, or withdrawing treatment, and the contextual factors involved.

Finally, a frequency analysis was performed to assess the distribution of case types and ethical issues. This involved counting how many times each category appeared across the dataset, allowing for a quantitative overview of the most common clinical scenarios and ethical dilemmas. Although descriptive in nature, this step supported the thematic findings by highlighting recurring patterns and the relative prevalence of different concerns, thereby strengthening the interpretation of the qualitative data.

#### 2.3.4. Emotion Classification and Statistical Analysis

Participants specified whether they experienced emotions during or after deliberation. The responses were grouped into thematic clusters, each containing emotions that shared similar characteristics. For example, emotions such as “sorrow” and “sadness” were grouped due to their shared negative valence and affective tone. This clustering process ensured that each category was internally consistent, meaning that emotions within the same group were meaningfully related and clearly distinct from the other clusters, thus enhancing the reliability of the analysis. The emotions and feelings were thus categorized as negative or positive/neutral following established frameworks (Cohn & Fredrickson, 2009; Cambria et al., 2012) and classified into four categories as follows:

- neg_emo_during: Negative emotions during deliberation;
- pos/neu_emo_during: Positive/neutral emotions during deliberation;
- neg_emo_after: Negative emotions after deliberation;
- pos/neu_emo_after: Positive/neutral emotions after deliberation.

Finally, a frequency analysis was conducted to examine the prevalence of each emotion category, providing an overview of emotional trends in ethical decision-making.

## 3. Results

### 3.1. Participants’ Description

Of the 52 consultants who responded, 63.8% were based in Europe, while 36.2% were from the United States. The respondents were from the U.S.A. and 10 European countries: France, Germany, Italy, Ireland, the Netherlands, Norway, Spain, Switzerland, and the United Kingdom. Approximately thirty subjects did not join the study for the following reasons: the e-mail was incorrect; no feedback or response was ever received; lack of interest in participating; no longer an active member of an ethics committee; language barrier. The scarce number of participants is also due to the fact that many European countries still do not provide an ethical consultation service.

Among the 52 CECs who completed the interviews, 64.3% were male, while 35.7% were female. Their ages ranged from 31 to 70 years, with the majority being between 51 and 60 years old, accounting for 36% of the participants. All consultants had advanced education in bioethics/medical ethics, philosophy, or medical/life sciences. Specifically, 48.6% held a Ph.D. Of the participants, 27% were physicians, including geneticists, oncologists, and psychiatrists, while 24.3% had graduate degrees in areas like nursing, social work, or theology, as well as bioethics and healthcare. Regarding professional experience, 60.5% had over 10 years in the field, 22% had between 5 and 10 years, and 17.5% had less than 5 years of experience as an ethicist.

### 3.2. Cases Selected and Primary Ethical Issues

The cases selected by participants were diverse and intricate, as detailed in Table 2. These data underscored the broad spectrum of medical scenarios encountered in hospitals, highlighting the difficulties faced by ethical consultants in achieving consensus among all stakeholders, including patients, healthcare providers, and families. These difficulties were especially notable when the patient was unable to communicate their wishes or when there was disagreement among the parties involved. Nearly half of the cases described by the interviewees (44.23%) involved terminally ill patients, covering various age groups, including minors. Participants did not concentrate on a specific age group but instead dealt with a range of situations involving infants, adults, the elderly, and terminally ill patients in oncology or psychiatry. Consequently, this cluster was designated “Terminally Ill Patients”. The second most frequently identified category (28.85%) was labelled “Multi-Complex Patients”, and included cases with patients suffering from multiple comorbidities, creating uncertainty for the medical team on the appropriate course of action.

Moreover, approximately 10% of the interviewees noted personal conflicts among team members (such as disagreements between physicians or other healthcare specialists) as a significant ethical issue. This cluster was named “Disagreement Within the Team”. Other reported categories encompassed ethical issues concerning patients in coma or vegetative states, preferences regarding DNR (Do Not Resuscitate) or CPR (Cardiopulmonary Resuscitation), non-medical abortions, euthanasia, and diagnostic errors. Additionally, an incident involving racial discrimination was also reported.

The ethical questions raised during consultations included requests for the suspension of care (withdraw, 30%), refusal of treatment (withhold, 11%), or both (14.5%). In other cases (30.4%), consultations were sought for various medical decisions, such as whether to proceed with surgery, abortion, or alternative treatments, whether to discharge a patient, and how to proceed in general. Consultations were also requested to resolve disagreements within the healthcare team and address a case of racial discrimination. Finally, there was an instance where family members asked to keep an ominous diagnosis secret from the patient.

### 3.3. Emotions or Feelings Experienced During the Case Deliberation

The emotions and feelings experienced by consultants during case deliberations were categorized into negative and positive/neutral. Surprisingly, 76,7% of the subjects reported predominantly negative emotions, while only 21% also mentioned experiencing positive or neutral emotions. A small fraction (2.3%) claimed not to have felt anything. Table 3 illustrates the spectrum of emotions or feelings reported by the participants. Among negative emotions, the most frequently mentioned were frustration (22.73%) and sadness or sorrow (18.2%), followed by anger or irritation (16.67%). Other emotions, such as insecurity or confusion (9.1%), fear (6%), and distress (6%), were less cited but provided valuable insights into the consultants’ experiences. However, despite the circumstances, consultants also reported experiencing emotions of sympathy and humanity towards the patients and their families. In this context, the most frequently mentioned positive emotions or feelings were commitment or responsibility (39.9%), empathy or compassion (33.3%), and pride (11.1%).

### 3.4. Emotions or Feelings Experienced After the Case Deliberation

In the aftermath of deliberating ethical cases, 46.5% of subjects reported experiencing positive/neutral emotions or feelings, while 42.3% continued to experience negative emotions. Additionally, 7% indicated feeling nothing, and 4.2% did not respond to the question. Notably, those who reported negative emotions noted a decrease in their intensity compared to during deliberation. According to Table 4, the most reported were feelings of satisfaction (39.4%), peace or relief (33.3%), and commitment (12.1%). The most frequently cited negative emotions included frustration (33.3%), sadness or sorrow (20%), anger (20%), helplessness (13.3%), concern (6.7%), and solitude (6.7%), indicating a potential need for discussion or support.

### 3.5. Experience of Regret

The first open-ended question regarding “regret” prompted participants to reflect on their past actions, revealing that 45% of them felt a sense of discomfort or acknowledged they could have acted differently if given the chance. For example, one participant wrote, “Looking back, I would have handled the situation differently”, while another noted, “I would have scheduled additional meetings with the patient’s family to ensure everyone’s concerns were heard and to avoid misunderstandings”. Conversely, 55% expressed confidence in managing the situation well from start to finish. In the second question, which directly asked about regrets, 22% of participants admitted to experiencing regret or remorse, highlighting a significant decrease compared to the broader sense of unease expressed earlier (33% fewer respondents). The majority, 78%, reported no regrets, and when asked if they would confirm their decision again today, 80% of consultants affirmed they would. However, 12% expressed uncertainty, suggesting they might reconsider their decision (“perhaps” or “maybe” were the typical answers to this open question). A smaller percentage, 5%, openly stated they would change their decision in hindsight, while one participant did not respond.

## 4. Discussion

Research in cognitive psychology and decision-making has extensively demonstrated the influence of emotional experiences on the process of making inferences and decisions (e.g., Gawronski et al., 2018; Greene et al., 2001; Greene, 2005; Helion & Ochsner, 2018; Marques et al., 2023; etc.). This influence extends to how decisions are framed, the options considered, and the final choices made. The concept of affective heuristics further highlights how emotional states can profoundly shape judgments and choices, often leading individuals to rely on feelings rather than comprehensive analysis (Whelehan et al., 2020). Similarly, the “affect-as-information” theory affirms that people rely on their emotions as a key source of guidance for making judgments and decisions (Gangemi et al., 2021; Schwarz, 2012; Schwarz & Clore, 1983). Additionally, it is crucial to recognize that moral decision-making evolves from early childhood and is influenced by various factors such as cognitive growth, gender, personality traits (Friesdorf et al., 2015; Reber & Tranel, 2017; Sedlár & Gurňáková, 2025), and religious or spiritual beliefs (Szekely et al., 2015). Social norms—whether formal or informal, personal or collective, and descriptive or prescriptive—also significantly impact this process (Bicchieri, 2016).

While extensive research has explored how these factors shape moral decision-making, including in healthcare, there has been less focus on the emotional influences on CECs’ reasoning abilities. Instead, much of the research on CECs has concentrated on the role of heuristics and biases (e.g., Magelssen et al., 2014; Pedersen et al., 2009a, 2009b; Räsänen, 2023). The exploratory data presented here provide a valuable foundation for future research to deepen understanding of the relationship between emotion and moral reasoning in CECs. This study highlights a critical yet underexplored dimension of clinical ethics consultations by shedding light on the emotional undercurrents that shape ethical deliberations. A nuanced understanding of CECs’ emotional experiences is thus essential for generating informed hypotheses and designing targeted research tools that capture the affective dynamics of moral decision-making.

### 4.1. Emotions and Feelings in CECs: Implications and Future Directions

Participants frequently experienced negative emotions during and after case deliberation, supporting the documented link between moral dilemmas and negative emotions (Avramova & Inbar, 2013; Horne & Powell, 2016; Spronk et al., 2017, 2022). However, positive or neutral emotions such as commitment, empathy, and compassion were reported during deliberation, while post-deliberation emotions shifted toward satisfaction, peace, and relief, aligning with Spronk et al. (2022).

Nearly half of the participants also reported feelings of reconsideration about their approach to ethical situations, though the term “regret” was intentionally omitted from the first question to avoid bias. This initial open-ended framing may have elicited a broader spectrum of negative emotional responses, such as unease, sadness, or cognitive dissonance, that are often intertwined with, or may implicitly contain, the experience of regret or remorse. When the term regret was later introduced directly, fewer participants acknowledged it, possibly due to its greater psychological and moral weight. Regret—and related moral emotions like guilt—typically entail critical self-reflection, personal accountability, or a perceived moral shortcoming (Boyle et al., 2020). These aspects make such emotions more difficult to acknowledge, even anonymously. It is therefore plausible that the earlier expressions of unease or sadness may function as precursors or less threatening proxies for regret. This hypothesis is consistent with findings that people may avoid labeling emotionally intense states due to their potential impact on self-image (Boyle et al., 2020). Research further suggests that individuals may experience a vague sense of “being wrong” or cognitive discomfort, known as “Feeling of Error” (FOE), without explicitly recognizing regret or the error made (Gangemi et al., 2015). Conversely, the “Feeling of Rightness” (FOR) occurs when an intuitive response is followed by metacognitive certainty (Prowse Turner & Thompson, 2009). The observed discrepancy thus suggests that while participants more readily disclosed general emotional discomfort, they may not have fully recognized or acknowledged the feeling of regret, underscoring the complexity and sensitivity of moral emotions in ethical decision-making. Although the FOE questionnaire was not utilized in this study, its relevance for future research is acknowledged. Indeed, some CECs indicated finally, in the third question, that they would reconsider their final decision, suggesting that incorporating FOE assessments in future studies could enhance understanding of the intuition’s role in ethical decision-making. These findings further highlight the complex interplay between cognitive processes and emotional responses in ethical decision-making, warranting further investigation into subconscious influences on judgment. This is particularly relevant when ethics consultants’ decisions are final or highly influential in medical settings. If ethics consultants experience remorse or FOE, it suggests that their judgment may have been compromised at the time, potentially due to situational factors that disrupted their focus. After gaining emotional and cognitive distance, they may choose different approaches in similar future cases.

Furthermore, this study underscores the impact of cumulative negativity during ethical deliberations. Prolonged exposure to moral distress may contribute to stress-related conditions such as burnout, addictions, psychosomatic symptoms, impaired attention, insomnia, depression, and anxiety, all of which can significantly affect ethics consultants’ reasoning and decision-making abilities (Gawronski et al., 2017; Hermans et al., 2017). While frustration was the most reported emotion, some participants also experienced anxiety and distress. However, it is essential to distinguish among different levels of stress—low, acute, and chronic—as moderate stress can be beneficial for cognitive performance (McEwen, 2019). In contrast, elevated stress impairs cognitive control, reduces confidence in decision-making, and diminishes the inclination to act (Gawronski et al., 2017; Hermans et al., 2017). Chronic stress, in particular, negatively impacts the prefrontal cortex, a brain region critical for complex cognition, personality traits, decision-making, and social interactions. This impairment may result in more automatic and less reflective responses (Arnsten, 2009; Dias-Ferreira et al., 2009). This finding thus raises questions about the coping strategies CECs employ to manage the emotional toll of patient illness or death—an acknowledged challenge in healthcare (Chen et al., 2019; Dahò, 2020). Further research is thus needed to explore how CECs navigate and regulate their emotions in ethically complex scenarios.

In addition, these findings underscore the importance of addressing the emotional burden associated with ethical deliberation to support the well-being and decision-making effectiveness of CECs. A consultant emotionally affected by a previous complex case might, for instance, adopt an overly cautious stance and recommend conservative treatments, even when more proactive interventions would align better with the patient’s values and medical needs. Alternatively, lingering emotional tension could lead to a more aggressive approach, pushing for interventionist decisions to regain control or resolve internal moral conflict. Future research should thus focus on developing strategies to mitigate stress and enhance resilience among these specialists. Furthermore, studies in neuroscience and psychopathology can provide valuable insights into how cognitive and emotional processes influence ethical reasoning, thereby informing the development of more effective training and support strategies (Falzone et al., 2023). Many scholars have indeed highlighted the absence of standardized training programs for CECs, which often fail to equip them with the essential knowledge, skills, and ongoing support needed for their roles (e.g., Fox, 2016; Lipman & Powell, 2016; Ong et al., 2020). These programs also neglect key socio-cultural, research, and mental health factors. To address these gaps, strategies such as providing access to psychological resources, implementing regular emotional resilience training (e.g., Gangemi et al., 2019; Murden et al., 2018; Smith & Ascough, 2016), and cultivating a work environment that acknowledges the emotional challenges of ethical decision-making are crucial.

Such measures are essential not only for safeguarding the well-being of CECs but also for ensuring the quality and integrity of healthcare decisions. The risk of suboptimal or biased recommendations may increase when ethical deliberations are impaired, due to emotional overload, inadequate support, or burnout. These flawed decisions can, in turn, negatively affect patient care, leading to outcomes such as delayed treatment, inappropriate care plans, or erosion of trust in the healthcare system. For instance, poor ethical judgments and bias have been linked to increased patient morbidity and dissatisfaction with care pathways (Preisz, 2019; Dahò et al., 2025). Conversely, well-founded ethical guidance can lead to markedly better outcomes. For example, informed decision-making and health worker support in perinatal hospices have been shown to enhance parental satisfaction, bolster family psychophysical well-being, and facilitate healthier grief processing (Dahò, 2021, 2024). To conclude, addressing the emotional and psychological demands placed on CECs is crucial for fostering resilient and ethically sound consultation practices, including during medical emergencies (Simon, 2016; Tarzian, 2024).

### 4.2. Strengths and Limits of the Study

This study fills an important gap in understanding the emotional experiences of CECs during and after case deliberations, providing valuable insights into this relatively underexplored area and paving the way for future investigation. Its findings have implications for clinical and medical ethics consultation, potentially guiding the creation of support systems and training programs designed to alleviate the adverse effects of stress and emotions on decision-making processes. One of the study’s strengths is its use of open-ended questions, which allowed for a valuable examination of consultants’ experiences and feelings. However, the limited sample size restricts the generalizability of the results. Additionally, as qualitative data are self-reported, they may be subject to biases such as social desirability or retrospective recall. The study’s design also does not establish causal relationships between emotional experiences and decision-making over time, highlighting the need for further investigation. Despite these limitations, examining personal experiences represents a crucial initial step toward establishing a foundation for future research in this domain.

## 5. Conclusions

Moral dilemmas, especially those involving intricate medical cases and ethical conflicts, elicit strong emotional responses from consultants. This highlights the demanding nature of clinical ethics consultations and the critical impact of decisions made in these situations. Emotions such as frustration, sadness, and anger experienced during deliberations can influence decision outcomes and lead to subsequent feelings of regret. Combined with consultants’ reflections on their decisions post-deliberation, these findings point to the need for a deeper investigation into how emotions affect decision-making processes in this context. Additionally, the accumulation of negative emotions over time can negatively impact consultants’ mental health, potentially resulting in stress-related disorders. Addressing these consequences is vital for ensuring consultants’ effectiveness and overall well-being. Overall, the study emphasizes the importance of understanding and managing the complex interplay between cognitive and emotional processes in medical ethics consultations, with significant implications for consultants and the quality of healthcare decision-making.

## Figures and Tables

**Table 1:** Survey questions.

**Demographic questions:**	Age (write the number); Gender (Open question); Country of Origin (Open question); Highest level of education and major field (Open question); How long have you been working as a CEC in any healthcare organization? (Options: 1–5 years; 6–10 years; +10 years);
**Case questions:**	Could you describe briefly an ethical clinical case that you perceived as challenging? (Please include your final decision and dismiss the patient’s personal information); What do you consider the primary ethical issue or dilemma raised by the situation? How did you face this clinical case? Did you follow a standard approach based on literature or a free form?
**Emotional experience questions**:	What emotions or feelings did you experience during the deliberation and discussion of the case? What emotions or feelings do you feel now after the deliberation? Did you ever get the feeling that you did not handle the situation correctly? If so, what would you have done differently? Do you ever feel regret about this case? If yes, can you explain what you regret and why? Would you confirm or change your decision now?

**Table 2:** Cases selected and primary ethical issues.

	Frequency	Percentage
Terminally_ill_patient	23	44.23
Multicomplex_patient	15	28.85
Disagreement_Team	5	9.62
Coma	3	5.77
Abortion_NoMed_Reason	2	3.85
Misdiagnosis	1	1.92
Euthanasia	1	1.92
DNR_CPR	1	1.92
Racial_Discrimination	1	1.92
**Total**	52	100.0

**Frequency**

**Percentage**

**Table 3:** Emotions or feelings experienced during deliberation.

Negative Emotions/Feelings			Positive/Neutral Emotions/Feelings		
	Frequency	Percentage		Frequency	Percentage
Frustration	15	22.73%	Commitment/ Responsibility	8	38.9%
Sadness/sorrow	12	18.2%	Empathy/ Compassion	6	33.3%
Anger/Irritation	11	16.67%	Pride	2	11.1%
Insecurity/ Confusion	6	9.1%	Curiosity	1	5.6%
Fear	4	6%	Interest	1	5.6%
Distress	4	6%	Excitement	1	5.6%
Helplessness	3	4.55%			
Concern	3	4.55%			
Anxiety	3	4.55%			
Disappointment	3	4.55%			
Regret	2	3%			
**Total**	66	100.0	**Total**	18	100.00

**Table 4:** Emotions or feelings experienced after deliberation.

Positive/Neutral Emotions/Feelings			Negative Emotions/Feelings		
	Frequency	Percentage		Frequency	Percentage
Satisfaction	13	39.4%	Frustration	10	33.3%
Peace/Relief	11	33.3%	Sadness/sorrow	6	20%
Commitment	4	12.1%	Anger	6	20%
Compassion	3	9.1%	Helplessness	4	13.3%
Curiosity	1	3%	Concern	2	6.7%
Pride	1	3%	Solitude	2	6.7
**Total**	33	100.0	**Total**	30	100.0

## Data Availability

The data are not available to protect the confidentiality and anonymity of the participants. The cases described in this study are unique and involve rare clinical situations. Given the specificity of these cases, there is a risk that individuals—whether patients or members of the medical team—could be identified based on the details provided. To prevent any potential re-identification, all data will remain strictly confidential.

## Abbreviations

The following abbreviations are used in this manuscript:

CECs	Clinical Ethics Consultants
DNR	Do Not Resuscitate
CPR	Cardiopulmonary Resuscitation
FOE	Feeling of Error
FOR	Feeling of Rightness
